# Sleep Disturbance and Perimenopause: A Narrative Review

**DOI:** 10.3390/jcm14051479

**Published:** 2025-02-23

**Authors:** Libera Troìa, Martina Garassino, Agnese Immacolata Volpicelli, Arianna Fornara, Alessandro Libretti, Daniela Surico, Valentino Remorgida

**Affiliations:** 1 Department of Gynaecology and Obstetrics, University Hospital Maggiore della Carità, 28100 Novara, Italy; martinagarassino@gmail.com (M.G.); sissi.volpicelli@gmail.com (A.I.V.); arianna.fornara@gmail.com (A.F.); libretti.a@gmail.com (A.L.); daniela.surico@med.uniupo.it (D.S.); 2 Department of Translational Medicine, University of Eastern Piedmont, 28100 Novara, Italy

**Keywords:** sleep disorders, sleep disturbance, insomnia, perimenopause, menopausal transition

## Abstract

**Background/Objectives**: Perimenopause, impacting 80–90% of women, encompasses a range of vasomotor, urogenital, cognitive, and psychiatric symptoms associated with the fluctuation and gradual reduction of gonadal hormones. Moreover, the onset or worsening of sleep disturbances is prevalent during the menopausal transition. This narrative review seeks to elucidate the pathogenetic processes behind sleep disturbances during perimenopause and the main therapeutic options. **Methods**: The electronic databases PubMed, Scopus, Google Scholar, Web of Science, and Embase were queried for publications up to May 2024. Longitudinal, observational, case–control, and cross-sectional studies, as well as reviews and meta-analyses, were included in the review in order to explore the prevalence of sleep disorders during perimenopause, the pathogenetic mechanisms underlying the association between menopausal transition and sleep disorders, and the available non-pharmacological and pharmacological treatment options. **Results**: Sleep disturbances are common among perimenopausal women and include insomnia, sleep-related breathing disorders, and movement disorders. Fluctuations in estrogen and progesterone affect sleep quality, while vasomotor symptoms can disrupt sleep. Circadian changes, decreased melatonin production, and physiological changes associated with aging and mood disorders further exacerbate sleep disturbances. **Conclusions**: Managing sleep disorders in perimenopause requires an individualized approach, considering the multifactorial nature of these disturbances and providing background knowledge about the relationship between reproductive hormonal changes and sleep. Non-pharmacological treatments should be considered the first-line therapy; hormone therapy or non-hormonal pharmacological treatments can be considered according to the patients’ specific needs and risk factors. However, there is still a lack of standards on the appropriate management and treatment of sleep disorders in perimenopause.

## 1. Introduction

The Stages of Reproductive Aging Workshop (STRAW) criteria divide the female life into three phases: reproductive phase, menopausal transition phase, and postmenopausal phase [1].

Perimenopause, or menopausal transition, is a period of progressive reduction in ovarian function, resulting in fluctuating levels of gonadal steroids such as estrogen, progesterone, and testosterone. This leads to irregular menstrual cycles, anovulatory cycles, and a wide variety of symptoms affecting multiple organ systems [2]. Eighty to ninety percent of women experience symptoms of menopause and perimenopause. Many women during menopausal transition complain of hot flushes and night sweats, altered moods, and vaginal and sexual changes, as well as urogenital symptoms like vulvovaginal atrophy, dryness, dysuria, polyuria, incontinence, and dyspareunia. Psychological and cognitive disorders are also frequently associated, including depression, anxiety, and a higher risk of dementia [3].

The new onset or worsening of sleep disorders is common in the menopause transition.

More broadly, women are more likely than men to report insomnia and nonspecific symptoms of sleep apnea, such as fatigue or mood disturbances. These differences are influenced not only by hormonal fluctuations throughout a woman’s reproductive life—such as during pregnancy and menopause—but also by biological and behavioral factors. Women are more frequently affected by sleep disorders such as restless leg syndrome (RLS), sleep apnea, and narcolepsy, often experiencing more severe or atypical symptoms compared to men. For instance, women with sleep apnea may not exhibit the classic signs of snoring and excessive daytime sleepiness but instead report fatigue, exhaustion, or mood disturbances, which can lead to delayed diagnosis. Additionally, sleep disturbances are more prevalent in women during pregnancy and menopause due to anatomical and physiological changes, such as weight gain and fluid retention, which contribute to airway collapsibility [4,5].

The prevalence of sleep disorders ranges from 16% to 47% during the perimenopausal phase and increases to 35% to 60% in menopause [4]. Frequent and early awakenings, trouble falling asleep, and interrupted sleep are hallmarks of sleep disorders in the menopausal transition. Insomnia, sleep apnea, periodic limb movement, restless leg syndrome, and the incidence of nocturia increase after menopause [5].

Both estrogen and progesterone are positively associated with sleep during the menopausal transition. Indeed, perimenopausal hormone therapy induces a marked improvement in sleep disturbances. However, other causes of sleep disturbances, such as aging, changes in stress hormones metabolism, the secondary effects of vasomotor symptoms, or depression, must be considered [6].

Perimenopause can last up to four years before the last menstruation, and the effect of the symptoms on quality of life can be significant. In addition, in patients suffering from chronic sleep disorders, emotional well-being and physical functioning may be significantly impaired, and long-term consequences for mental and physical health may occur [7].

Managing these concerns can be challenging for clinicians because symptoms can affect each other, and improper management can exacerbate the condition [7]. Therefore, appropriate treatment has an immediate benefit, as well as advantages for maintaining optimal health in the postmenopausal years.

Considering the multifactorial basis for sleep disorders in women transitioning menopause, this article aims to summarize the pathogenetic mechanisms at the basis of the association between menopausal transition and sleep disorders and the main therapeutic options.

## 2. Materials and Methods

The main electronic databases PubMed, Scopus, Google Scholar, Web of Science, and Embase were searched for literature published up to May 2024. The authors aimed to include in the review any studies investigating the prevalence of sleep disorders in menopausal transition; the pathogenetic mechanisms at the basis of the association between menopausal transition and sleep disorders; and the non-pharmacological and pharmacological treatment options. Several combinations of terms were used to query the databases. These included “sleep disorders”, “sleep disturbance”, “sleep quality”, “sleep duration”, “insomnia”, “insomnia symptoms”, “sleep-related breathing disorder”, “narcolepsy”, “hypersomnia”, “obstructive sleep apnea”, and “perimenopause”, “menopausal transition”, or “menopause transition.” Studies were included if they (1) involved human adults; (2) were longitudinal, observational, case–control, or cross-sectional studies, reviews, or meta-analyses; and (3) were published before May 2024. Studies were excluded if they were (1) not available in full text or (2) in a language other than English.

The first two authors (L.T. and M.G.) extracted the data independently. To better summarize the findings, and due to the nature of the topic, the data were integrated into a narrative review. This approach allowed us to explore a wider range of aspects related to sleep and perimenopause, offering a more comprehensive assessment.

## 3. Results

### 3.1. Sleep Disorders and Perimenopause

Sleep disturbance is a very common issue among perimenopausal women. In the Study of Women’s Health Across the Nation, 37% of women aged 40–55 years reported difficulty sleeping, with higher rates in Caucasian and Hispanic women [3,8].

Poor sleep quality, especially post menopause, and related hormonal changes are risk factors for cardiovascular disease, obesity, diabetes, psychiatric disorders, and an increased mortality risk [8,9]. An independent relationship between sleep disorders and menopause, beyond confounding factors like physical problems (e.g., lower back pain, musculoskeletal disorders) and menopause-related symptoms like hot flushes and urinary issues, was demonstrated [10]. Premenopausal sleep condition is a strong predictor of sleep disturbances throughout the menopause transition phase [8]. Sleep symptoms tend to stabilize with the transition to postmenopause [8].

Sleep habits should be routinely assessed during clinical evaluations because of their significant impact on quality of life and health outcomes. The primary cause of sleep disturbance and other associated clinical conditions should be identified.

Objective (polysomnography) and subjective (self-assessment tools or questionnaires) methods for sleep assessment can be used. Screening for psychiatric disorders is required before establishing that hormonal changes are the main cause of perimenopausal sleep disorders.

The International Classification of Sleep disorders (ICSD-3) divides sleep disorders into seven major categories: insomnia, sleep-related breathing disorders, central disorders of hypersomnolence, circadian rhythm sleep–wake disorders, sleep-related movement disorders, parasomnias, and other sleep disorders [11]. Each major category is divided into subsections.

The most prevalent sleep disorders, with regard to their potential association with female reproductive hormones, are insomnia, sleep-related breathing disorders, and sleep-related movement disorders [6].

#### 3.1.1. Insomnia

Insomnia is defined as a quantitative or qualitative dissatisfaction with sleep according to the *Diagnostic and Statistical Manual of Mental Disorders*, Fifth Edition (DMS-5). It may be associated with one or more of the following features: difficulty falling asleep, difficulty maintaining sleep, and morning awakening with inability to resume sleep [12]. This results in daytime impairment, despite adequate opportunity and circumstances to sleep. With an overall prevalence of 22%, insomnia is considered the most common sleep disorder [7,10]. It can be classified as acute, usually related to a temporary stress-related condition, or chronic if it lasts more than three months [7,10]. Women are 1.5 times more likely to experience insomnia than men. Aging, depressive symptoms, and high stress levels are other risk factors for insomnia occurrence.

#### 3.1.2. Sleep-Related Breathing Disorders

There are distinct types of abnormal breathing during sleep, obstructive sleep apnea (OSA) and central sleep apnea, with the latter involving the dysregulation of breathing neural centers (e.g., narcolepsy and hypersomnia) [13]. Sleep-related breathing disorders occurs in 70% and 56% of elderly men and women, respectively [13]. Although men are generally more affected by sleep-disordered breathing than women, the prevalence increases when women reach menopause. Obesity is a known risk factor, but hormones are also involved in the patho-physiology of this sleep disturbance [13].

#### 3.1.3. Sleep-Related Movement Disorders

Restless leg syndrome (RLS) and periodic limb movement disorder (PLMD) are classified as sleep-related movement disorders according to DMS-5 [12]. RLS is characterized by a continuous impulse to move the legs, most frequently during the night. Women are affected about twice as much as men (9% vs. 5%). PLMD is another sleep disturbance in which patients unintentionally move their extremities during sleep. It is common in the elderly, especially in women, but can also occur in pregnancy [14]. Often, the two disorders, RLS and PLMD, occur together [6].

### 3.2. Pathophysiologic Links

Perimenopausal sleep disturbances are multifactorial, representing a key symptom of women’s transition phase. Physiological changes, lifestyle factors, and menopausal symptoms such as night sweats, nocturia, and urinary incontinence are involved, as well as ethnic and socioeconomic factors. Mood disorders such as anxiety and depression are strongly associated.

#### 3.2.1. Hormonal Changes

An overall interplay between the reproductive and sleep systems has been demonstrated. As women are more affected by sleep disorders than men, and sleep disorders are most prevalent during periods of hormonal shifts, such as pregnancy or menopausal transition, changes in reproductive hormones are involved in sleep rhythm regulation [6].

Perimenopause is characterized by irregular menstrual cycles due to fluctuations in sex hormones. With aging, ovarian reserves decrease, estrogen fluctuates through lower levels, progesterone progressively decreases, and AMH and inhibin A and B levels decrease. Due to a negative feedback mechanism, FSH and LH increase [3]. Decreases in inhibin B and increases in FSH are typically early changes preceding decreases in estradiol and inhibin A in late menopausal transition [9].

In addition, the decrease in ovarian hormones and the increase in sex hormone-binding globulin (SHBG) also lead to a relative increase in androgens. Besides reproductive hormones, stress hormones also increase with age and impact on sleep [3].

Both estrogen and progesterone have a favorable influence on subjective sleep quality and total sleep duration, with a more rapid sleep onset and fewer awakenings. Direct or indirect, peripheral or central hormonal action is involved [15].

##### Estrogen

Estrogen is a neuroactive steroid affecting sleep regulation through multiple and complex pathways. A direct action on sleep regulatory nuclei, the preoptic area of the hypothalamus, and suprachiasmatic nucleus is well known. On the other hand, through an indirect effect, estrogen influences other neurotransmitter systems involved in sleep regulation, including dopaminergic and serotonergic systems [15]. Estrogen inhibits neurotransmitters involved in awakening, such as acetylcholine, histamine, noradrenaline, serotonin, orexin, and dopamine, thereby decreasing sleep latency and the number of awakenings [15]. Furthermore estrogen may act on the thermosensory pathway and the thermoregulatory nuclei in the hypothalamus, in order to modulate body temperature, helping to maintain deep sleep phases [16].

In a recent systematic review by Haufe et al., the majority of included good-quality studies confirmed the association between lower estradiol concentrations and impaired sleep quality [15]. In contrast, a higher level of urine metabolite estrone 3-glucuronide was found in women with less severe night and morning awakenings [17].

Polysomnographic measurements have demonstrated that low estradiol is associated with sleep-disordered breathing, a high frequency of movement arousals, and lower sleep efficiency. A subjective sleep evaluation obtained by questionnaires found significantly lower levels of estradiol in women with insomnia, poor sleep, and difficulty staying asleep and falling asleep [6].

Furthermore, some authors stated that the degree and the dynamics of estrogen fluctuation, rather the absolute hormone level, was strongly associated with sleep disorders [18].

Conversely, others studies using subjective measurements found no association between sleep and estrogen [19].

##### Progesterone

Similar to estrogen, progesterone is also a neuroactive steroid, able to directly influence sleep and circadian rhythmicity through its receptors located in the sleep regulatory nuclei, including the basal forebrain, dorsal raphe nucleus, locus ceruleus, and SCN [20].

Progesterone has a sedative, anxiolytic, and hypnotic effect, mediated via its neuroactive metabolites, including allopregnanolone and pregnanolone, acting on GABA [9] and benzodiazepine receptors [20].

A negative correlation was found between allopregnanolone and sleep disorders in midlife women [21]. In contrast, one study found that pregnanediol glucuronide correlated with more trouble sleeping in perimenopausal women [22].

Based on actigraphy, perimenopausal women were found to have a lower sleep efficiency, shorter sleep time, and more variability in activity during sleep in the late luteal phase of the menstrual cycle when progesterone levels are declining, although hormonal levels were not assessed [23]. A small study found more polysomnography awakenings/arousals in the luteal versus the follicular phase in perimenopausal women with ovulatory cycles; however, relationships with progesterone were not examined. Progesterone stimulates respiration through a central steroid receptor-mediated mechanism and may also have a direct peripheral effect on upper airway dilator muscle activity [24]. In fact, progesterone has a relaxing effect on smooth muscles, both those of the upper airway and those of the lower esophageal sphincter contributing to the pathogenesis of gastroesophageal reflux [24].

This explains how progesterone therapy, via airway relaxation, improves sleep-disordered breathing in peri- and postmenopausal women. The effect on thermoregulatory centers represents another recognized mechanism of sleep regulation by progesterone.

#### 3.2.2. Vasomotor Symptoms

Vasomotor symptoms (hot flashes, night sweats) affect the majority of women undergoing the menopause transition, with a significant impact on sleep quality [10]. Hot flashes, characterized by a sudden rise in body temperature and vasodilatation, resulting in a flushing sensation, are likely to be significant contributors to nocturnal awakenings. These symptoms can last for 4–5 years, and sometimes persist for up to 10 years [10].

Hot flashes result from dysfunction of the hypothalamic thermoregulatory center due to estrogen decrease. Estrogen deprivation leads to increased norepinephrine and decreased serotonin blood levels. Moreover, estrogen withdrawal may affect the neural circuits that control temperature, through neurokinin B signaling in the arcuate nucleus of the hypothalamus [16].

A bidirectional relationship between vasomotor symptoms and sleep disorders has been widely demonstrated [25]. Nighttime hot flashes disrupt sleep, and women with more frequent and severe symptoms are more likely to report insomnia. Most flushes, measured objectively, coincide with awakenings, although it is unclear whether there are times differences in this relationship [10].

#### 3.2.3. Mood Disorders

Perimenopause is associated with an increased risk of mood disorders. A higher risk of new-onset depressive disorder during menopausal transition, even in women without prior history, has been demonstrated [26].

The menopausal transition significantly impacts quality of life, necessitating routine assessment for depressive disorders. In the SWAN cohort, psychological distress prevalence was 28.9% in early menopausal transition, 25.6% in late transition, and 22% in postmenopause [26].

Depression is a risk factor for sleep disturbance, creating a “domino effect” where sleep disruption due to hot flashes or other symptoms leads to anxious thoughts during sleep time, further disturbing sleep and affecting daytime mood [27].

One RCT examined links between estradiol, depression, sleep disturbance, and hot flashes [28].

Among perimenopausal women, an improvement in mood disorders is predicted by improved sleep quality and increased estradiol levels following estrogen therapy. These results support that changes in estradiol and sleep quality, rather than hot flashes, moderate depressive symptoms during the menopausal transition [28].

Furthermore nighttime, but not daytime, vasomotor symptoms contributed to a depressed mood independently of their effect on sleep in estrogen-deprived women [29].

The extent to which vasomotor symptoms are troubling or interfere with daily life predicts mood disturbances and quality of life more than vasomotor symptom frequency does [3].

Mood disorders, which can cause early morning awakenings, restless leg syndrome, and sleep apnea, should always be ruled out before attributing sleep disturbances to estrogen deficiency. The effective management of nocturnal vasomotor symptoms and sleep disturbances could play an important role in the prevention and management of mood disorders during the menopausal transition.

#### 3.2.4. Obstructive Sleep Apnea (OSA)

OSA is a chronic condition characterized by recurring incidents of upper airway obstruction during sleep, which increases the risk of cardiovascular disease due to hypoxia, oxidative stress, and sympathetic stimulation. Although more frequently diagnosed in men, OSA prevalence increases in perimenopausal women [30]. Weight gain and changes in fat distribution related to increased testosterone and decreased ovarian hormones explain the higher prevalence of OSA in menopause.

The incidence of RLS also increases. Sleep-disordered breathing is greater in obese postmenopausal women, and metabolic comorbidities contribute to the risk [30].

#### 3.2.5. Circadian Modifications

The hypothalamic suprachiasmatic nuclei and pineal gland are centers of sleep regulation. Epiphysis produces melatonin based on light levels. Age-related dysregulation in the circadian rhythm and melatonin production contribute to the development of insomnia. Since sex hormones contribute to the sensitization of neurons to melatonin, low estrogen levels are linked to reduced melatonin production and the onset of perimenopausal sleep disturbances [22].

### 3.3. Treatment of Sleep Disorders

Multifactorial etiopathogenesis makes the treatment of menopausal sleep disorders challenging. Tailored strategies must be identified based on the main symptoms experienced by women. Treatment options include non-pharmacological and pharmacological approaches. First-line treatment options often include psychological or behavioral therapies. Because of potentially serious health risks associated with the long-term use of hormone therapy (HT), it is important to consider non-pharmacological options or non-hormonal pharmacological alternatives; if these are not effective, HT should be proposed after an assessment of risk factors [31].

#### 3.3.1. Non-Pharmacological Treatment

Cognitive Behavioral Therapy for Insomnia (CBT-I), including sleep hygiene, stimulus control, sleep restriction, cognitive therapy, and relaxation training, is effective in treating insomnia during peri- and postmenopause.

A 2018 data analysis of four RCTs by Guthrie et al., comparing pharmacological and non-pharmacological treatments, demonstrated that CBT-I reduced insomnia symptoms and improved sleep quality more than pharmacological interventions. Therefore, CBT-I can be considered a first-line treatment in healthy middle-aged women with insomnia and moderate vasomotor symptoms [32].

However, economic costs and the limited availability of trained practitioners represent a limitation. A 2016 RCT conducted on 106 women aged 40 to 65 years reporting at least moderate insomnia symptoms and two or more daily hot flashes showed that telephone-delivered CBT-I or menopause education control (six telephone sessions over 8 weeks) improved sleep in peri- and postmenopause [33].

Light therapy is helpful in restoring a proper circadian rhythm and, together with exercise, has slight beneficial effects on total sleep duration and sleep performance, medium to small beneficial effects on sleep onset latency, and moderate beneficial effects on sleep quality. These adjuvant treatments can help patients improve sleep disorders [31].

#### 3.3.2. Hormonal Treatment Options

Hormonal pharmacological therapies are based on the role of sex hormones in the pathogenesis of sleep disorders. However, the findings regarding the effects of HT on objective measures of sleep are conflicting.

A 2017 systematic review by Cintron et al. highlighted that many studies lack proper screening for sleep disorders, making it difficult to determine the direct or indirect efficacy of HT on sleep disorders in perimenopause [34]. According to a recent meta-analysis, HT improved self-reported sleep outcomes but not sleep parameters on polysomnography [35].

The good-quality controlled clinical trials included in the systematic review by Haufe et al. demonstrated that combined HT led to an overall reduction in sleep disturbances [15]. Combined HT improved both objective and subjective sleep quality (relative to sleep latency, sleep efficiency, wakefulness after sleep onset, and sleep satisfaction), with deeper sleep and fewer awakenings during polysomnography [15].

The actions of estrogen and progesterone could be mediated by direct and indirect, central and peripheral hormonal effects, as well as other effects on mood and vasomotor symptoms. However, further RCTs, and studies examining neurobiological pathways as well, are needed [15].

Combined HT was also associated with better self-reported sleep measures, after controlling for vasomotor symptoms and depressive symptoms. A therapeutic effect could already be seen after two to three months of treatment and resulted in a long-lasting positive effect on sleep quality for more than three years [36]. An increased frequency of sleep disturbance occurs after the abrupt termination of HT.

Objective studies on the effects of estrogen treatment on sleep have shown mixed effects: some have found a reduction in wakefulness and sleep latency and an increase in REM sleep, while others have found no effect, despite positive results based on subjective measurements [15].

In an RCT conducted on 339 peri- and postmenopausal women with more than two troublesome hot flashes per day, treatment with low-dose oral estradiol or venlafaxine enhanced sleep quality and decreased insomnia complaints compared with a placebo [37].

Both estradiol and conjugated equine estrogens (CEEs) improved sleep quality, and the transdermal route was more beneficial than the oral route [31,38].

Progesterone treatment reduced sleep complaints, increased total sleep time, reduced wakefulness after sleep onset, and increased REM sleep [15]. Some authors found opposite results: specifically, higher progesterone levels were associated with more awakenings or arousals and a lower rate of slow-wave sleep [15,22,39].

A systematic review and meta-analysis of RCTs confirmed that micronized progesterone improves multiple aspects of the sleep cycle, particularly sleep onset latency, and self-reported sleep outcomes. Drowsiness is a documented adverse effect due to its sedative properties through binding to γ-aminobutyric acid A (GABA) receptors; therefore, nighttime administration is recommended to avoid impairing morning cognitive function [40].

Micronized progesterone reduced sleep disorders more than medroxyprogesterone acetate did, although both are useful [31].

The potential efficacy of HT to alleviate sleep disturbances should always be considered on balance with its negative side effects, such as thromboembolic events, and it should be evaluated whether the benefits outweigh the risks.

Additionally, recent studies show that transdermal treatment appears to be the safest type of hormone therapy in evaluating the risk of venous thromboembolism [41]; and micronized progesterone was more effective in improving sleep and reducing heart failure [40].

Although many studies have reported that sleep is improved with estrogen and/or progesterone/progestin supplementation, it is important to consider confounding factors, such as vasomotor symptoms, age, BMI, stressors, and ethnicity [15].

In addition, inconsistent findings regarding the association between sleep, ovarian hormones, and hormonal therapies can be explained by other methodological factors: study quality, differences in study groups, and assessment methods of sleep disorders, as well as differences in formulation, dosages, the route of administration, and the duration of hormonal therapy.

#### 3.3.3. Non-Hormonal Treatment Options

Non-hormonal treatments for sleep disorders include benzodiazepines, GABA agonists, dual orexin receptor antagonists, and other medications with a sedating effect (antidepressants, anxiolytics, antipsychotics, antihistamines). The occurrence of side effects, from residual daytime sleepiness to dependence, should be considered. Over-the-counter drugs and herbal therapies are also used to treat insomnia. However, the efficacy and safety profile of these substances are not well known. In addition, melatonin is useful for improving circadian rhythm disturbances without significant side effects [42].

##### Benzodiazepines and Z-Drugs

Z-drugs act as agonists of the benzodiazepine receptor component of GABA-A and are commonly used for treating insomnia. These medications are known to have relatively fewer side effects than benzodiazepines, as they primarily bind to type 1 GABA-A receptors and only produce a sleep effect. Several RCTs on Z-drugs have reported that zolpidem increases total sleep time, reduces wake time after sleep onset, and decreases the number of awakenings [43]. Zolpidem is currently the most commonly used drug for chronic insomnia, and the American Academy of Sleep Medicine also suggests using zolpidem in adults with sleep onset and maintenance disorders. Moreover, benzodiazepines are recommended as the main treatment for certain sleep disorders, such as REM sleep behavior disorder, restless leg syndrome, and periodic limb movement disorder, whereas in patients with sleep apnea, the use of benzodiazepines and Z-drugs can worsen the symptoms. Despite their usefulness, these medications are not considered first-line treatments for sleep disorders in perimenopause. Cognitive behavioral therapy should be attempted first, and if it proves ineffective, a combined pharmacological approach should be tried [43].

##### Melatonin

Melatonin supplementation improves insomnia symptoms and mood disorders in peri- and postmenopausal women without serious side effects. The effects of melatonin on sleep quality are consistent for any duration or dose, and there is no evidence of addiction or hangover, and only a slight potential for addiction [42].

Prolonged-release melatonin (PRM), designed to mimic an endogenous melatonin production pattern, is approved for insomnia in patients aged ≥ 55 years. The main significant and clinically relevant benefits are improved sleep quality and latency, morning alertness the next day, and quality of life. Responses may develop over several days [44]. An oral dose of 2 mg once daily for 3 months was generally well tolerated without rebound effects, withdrawal, or hangover effects, and with no safety issues in concurrent therapy with antihypertensive, antidiabetic, lipid-lowering, or anti-inflammatory drugs. Undesirable effects on cognition, memory, postural stability, and sleep structure were not observed with PRM [44].

Since PRMs do not act on GABA receptors, they have fewer side effects, such as cognitive decline, falls, rebound insomnia, dependence, tolerance, and withdrawal symptoms, compared to benzodiazepines or Z-drugs. Therefore, these drugs could be an effective alternative to conventional sleep medications in the elderly population, including peri- and postmenopausal women. However, further research is needed.

Ramelteon is a melatonin receptor agonist (MT1 and MT2) effective in improving sleep quality and efficiency, and it has been approved by the US FDA for the treatment of insomnia [45].

##### Antidepressants

Doxepin is the only tricyclic antidepressant approved by the United States Food and Drug Administration as a treatment for insomnia. Doxepin 3 mg or 6 mg significantly improved wake time after sleep onset, total sleep time, and sleep efficiency compared to a placebo control group, and a meta-analysis demonstrated its efficacy in treating sleep maintenance disorders [46].

##### Gabapentin

Gabapentin is widely known as one of the non-hormonal treatments for vasomotor symptoms in menopausal women. Yurcheshen et al. demonstrated that gabapentin, 300 mg three times a day, improved sleep quality in menopausal women with hot flashes compared to placebo-treated subjects, at 4 and 12 weeks [47].

##### Dual Orexin Receptor Antagonists (DORAs)

DORAs, which target the orexin signaling pathway, are involved in sleep–wake regulation and have the potential to effectively treat insomnia with fewer next-day residual effects than other sleep-promoting drugs with different mechanisms of action.

Recent findings demonstrated that the DORA suvorexant may be a well-tolerated and efficacious treatment of hot flash–associated insomnia in midlife women; however, the study duration was limited to 4 weeks [48].

The DORA lemborexant (LEM) is approved in several countries for the treatment for adults with insomnia and may be a potential treatment option for midlife women with insomnia. LEM is a competitive antagonist at orexin receptor types 1 and 2 and is thought to reduce wakefulness by attenuating the orexin-mediated wake drive. In the post hoc analysis of 280 midlife women enrolled in a large phase 3 trial of LEM for insomnia disorder, LEM may have provided benefits and was well tolerated, with consistent improvement in all sleep diary-based outcomes for up to 6 months [49].

## 4. Conclusions

Poor sleep quality is a significant public health concern, linked to mental health issues such as depression, anxiety, and mood disturbances, as well as physical conditions, including metabolic, cerebrovascular, and cardiovascular diseases. Insufficient sleep also affects cognitive function and productivity and increases the risk of accidents. Women experience higher rates of insomnia than men, with the prevalence increasing with age. Therefore, sleep disorders represent a key symptom of menopause transition. Known causes include the occurrence of vasomotor symptoms and an increase in mood disorders. However, many others factors, such as physiological alterations associated with aging, menopausal symptoms, poor health perception, and comorbidities, together with socioeconomic, psychosocial, and ethnic factors, are involved. An independent correlation between menopausal transition, hormonal changes, and sleep disturbance, beyond the effects of aging and other confounders, is evident.

Managing sleep disorders in perimenopause requires a comprehensive, individualized approach, considering the multifactorial nature of these disturbances.

Providing background knowledge about the relationship between female reproductive hormonal changes and sleep disturbances is highly clinically relevant and constitutes a challenge in patient care.

Teaching patients to recognize and metabolize changes in their bodies, accompanying them along a path that can be simple but also complex, is essential. Non-pharmacological treatments should certainly be favored; cognitive behavioral therapy should always be offered to these women; it is a useful tool in understanding what is happening to their body and learning to manage it.

Non-pharmacological treatments should be considered the first-line therapy; hormone therapy or non-hormonal pharmacological treatments can be considered according to the patient’s specific needs and risk factors.

However, there is still a lack of standards on the appropriate management and treatment of sleep disorders in perimenopausal women, and large-scale prospective studies are needed to further investigate the role of various treatments.

## Data Availability

Datasets are available through the corresponding author upon reasonable request.

## Abbreviations

The following abbreviations are used in this manuscript:

STRAW	The Stages of Reproductive Aging Workshop
ICSD-3	International Classification of Sleep disorders
DMS-5	Diagnostic and Statistical Manual of Mental Disorders, Fifth Edition
OSA	Obstructive sleep apnea
RLS	Restless leg syndrome
PLMD	Periodic limb movement disorder
FHS	Follicle-stimulating hormone
LH	Luteinizing hormone
AMH	Anti-Mullerian hormone
SHBG	Sex hormone-binding globulin
SCN	Central nervous system
GABA	Gamma-aminobutyric acid
HT	Hormone therapy
CBT-I	Cognitive Behavioral Therapy for Insomnia
RCT	Randomized controlled trial
BMI	Body mass index
PRM	Prolonged-release melatonin
DORA	Dual orexin receptor antagonist
